# Supplementary figures and images for: Network study of miRNA regulating traumatic heterotopic ossification
Source: PLoS One. 2025 Feb 11;20(2):e0318779. doi: 10.1371/journal.pone.0318779 (PMC11813146; doi:10.1371/journal.pone.0318779)

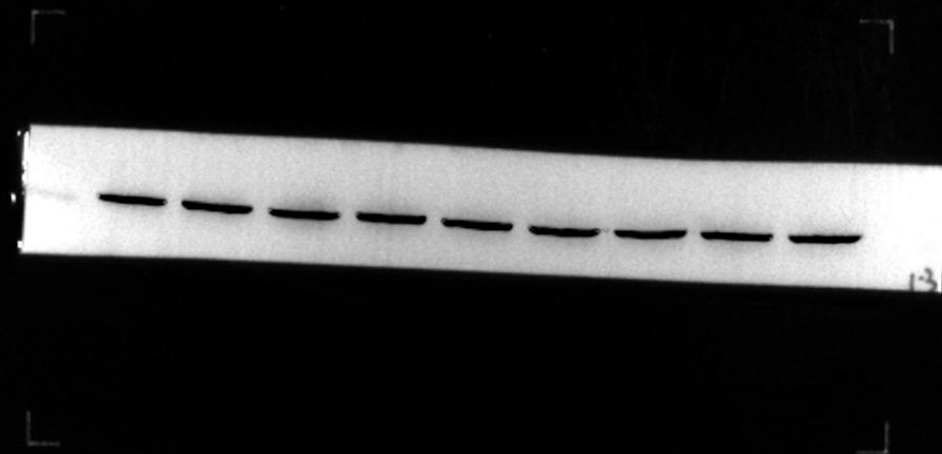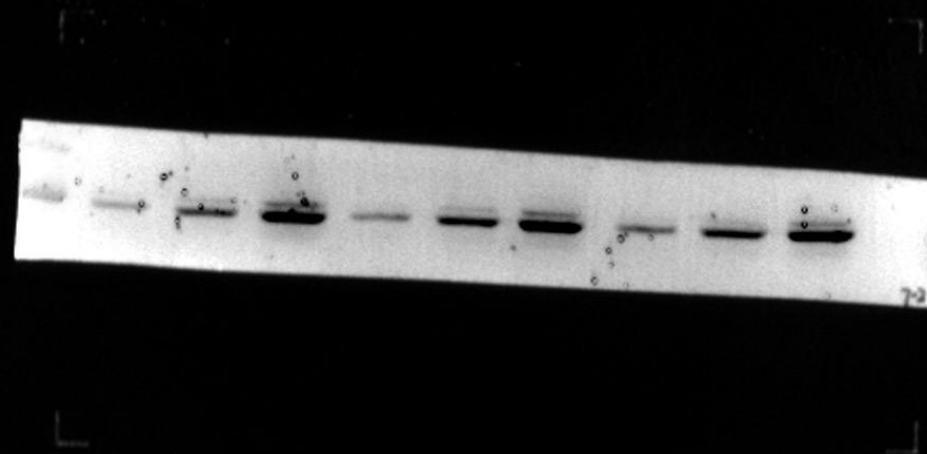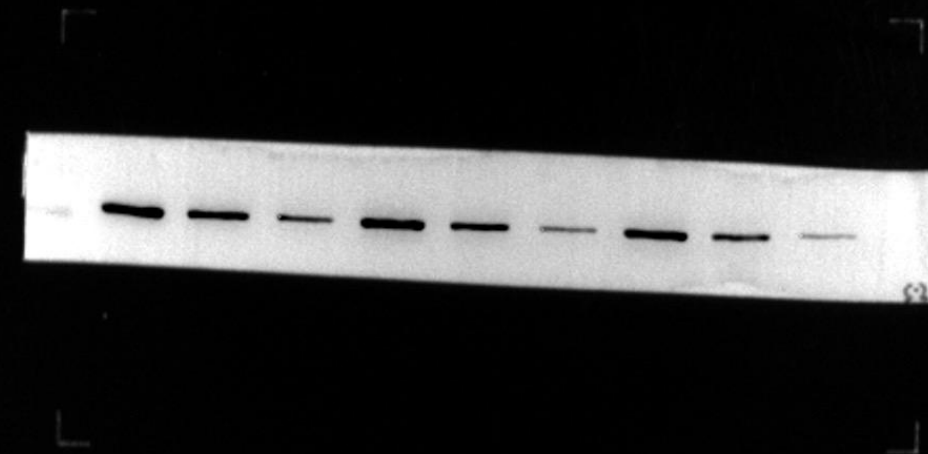

Supplement: S1 Raw images — (PDF) [file pone.0318779.s001.pdf]
