# Supplementary material for: Network study of miRNA regulating traumatic heterotopic ossification
Source: PLoS One. 2025 Feb 11;20(2):e0318779. doi: 10.1371/journal.pone.0318779 (PMC11813146; doi:10.1371/journal.pone.0318779)
Supplement: Attachment — Submitted filename: PONE-D-24-32971_review_comments.docx [file pone.0318779.s002.docx]

Review Comments for the manuscript “Network study of miRNA regulating traumatic heterotopic ossification”

The study aims to identify specific microRNAs differentially expressed in traumatic heterotopic ossification (THO) compared to normal bone formation. Tissue samples from THO and normal group patients were collected. RNAseq was used to identify differentially expressed miRNAs followed by GO and KEGG analysis to understand target pathways and further validated by RT-PCR. 87 DE RNAs were obtained (27 upregulated and 57 downregulated), and how they may be implicated in the development and therapeutic purpose are also discussed.

The manuscript is well-structured, reflecting the authors’ systematic approach to the investigation. However, a few points could be further clarified to ensure the manuscript is accessible to a wider audience.

**Question 1:**

The authors explain that “…a total of 14 patients who underwent surgical treatment in the Second Affiliated Hospital of Kunming Medical University were enrolled in this study, including 7 patients in THO group and 7 patients in the control group. … Three samples were chosen from each group for miRNA sequencing, while the remaining samples were utilized for subsequent verification tests”

The authors randomly selected three out of seven collected samples as the representative patient population for miRNA sequencing. Individual patient-to-patient variability may question the actual representativeness of the group. To address this issue of variability, future studies could consider a larger sample size or stratified sampling to account for potential variability.

**Suggestion 1:**

The authors have appropriately acknowledged the limitation of the sample size. They could further elaborate on how alternative approaches (i.e., Western blotting), could be used to strengthen the conclusion, in addition to increasing the sample size. This would demonstrate their awareness of potential research constraints and their commitment to addressing them.

**Suggestion 2:**

In line 374, the authors mention that they did not investigate any distinction from soft tissues. It would be beneficial to include a line or two explaining why comparing THO with soft tissues is crucial for understanding the specificity of the miRNAs to the pathology.

**Suggestion 3:**

The authors may add a few lines in the discussion section on the possible future experimental approaches that may be taken up in various in vivo and in vitro models to validate the predictive model results. Is it possible to highlight how the results of the current study may be used to develop therapeutic interventions in the future? This could inspire further research and motivate the audience to continue exploring this promising study area.
