# Supplementary material for: Network study of miRNA regulating traumatic heterotopic ossification
Source: PLoS One. 2025 Feb 11;20(2):e0318779. doi: 10.1371/journal.pone.0318779 (PMC11813146; doi:10.1371/journal.pone.0318779)
Supplement: Attachment — Submitted filename: Response to Reviewers.docx [file pone.0318779.s003.docx]

Dear Reviewer,

Thank you very much for your hard work and sincere guidance, and we sincerely appreciate your review of our manuscript.

Based on your valuable comments, we have revised the discussion section of the article and responded fully to the specific content, hoping that our modification can better compound the requirements of the article publication. If you have any unclear questions, please do not hesitate to contact me, we will reply in time.

Thank you again for your review.

We look forward to hearing from you soon.

Best wishes,

Lian

**Response to Suggestion 1:**

We thank the reviewers for their interest in the representativeness of our sample size. We acknowledge that differences between individual patients may affect the representativeness of our study group. To address this, as you suggest, we included additional analyses performed using alternative methods, such as Western blotting, to validate our findings. This approach provides a more comprehensive validation beyond the scope of RNA sequencing, thus strengthening our conclusions.

We added the following text to the "Discussion" section to reflect this: Given sample size limitations, we recognize the potential impact of patient differences on the results. To alleviate this problem, we used Western blotting to verify the expression of key proteins associated with differentially expressed mirnas. This additional layer of validation not only strengthens our findings but also demonstrates our commitment to addressing potential study limitations. Future studies with larger sample sizes and stratified sampling will be essential to further validate our results and explain differences in patient populations."

**Response to Suggestion 2:**

We understand the importance of comparing THO with soft tissues to understand the specificity of the miRNAs to the pathology. We have added the following explanation in the "Discussion" section to clarify the rationale behind this comparison:

"Comparing THO tissues with soft tissues is crucial for understanding the specificity of the miRNAs involved in the pathological process of heterotopic ossification. This comparison helps to differentiate the molecular signatures unique to THO from those present in normal soft tissues, which is essential for identifying potential therapeutic targets and biomarkers specific to THO."

**Response to Suggestion 3:**

We agree that discussing potential future experimental approaches would be beneficial for the readers. We have expanded the "Discussion" section to include a section on future directions, which outlines potential in vivo and in vitro models for validating our predictive model results and discusses the implications for therapeutic intervention development. The added text is as follows:

"The current study lays the foundation for future research by identifying differentially expressed miRNAs in THO. Future experimental approaches may include the use of various in vivo models, such as animal models of THO, to validate the role of these miRNAs in the development and progression of the disease. Additionally, in vitro studies using cell cultures derived from THO tissues and normal bone tissues could provide further insights into the mechanisms by which these miRNAs regulate osteogenic differentiation. These approaches will be crucial for translating our findings into potential therapeutic interventions, which could include the development of miRNA-based therapies targeting key pathways implicated in THO."

Based on your valuable guidance and in combination with the results of our research, we have revised Discussion section 4.4 Limitations and prospects 377-396 as follows:

In this study, we identified 84 DE-miRNAs that were differentially expressed in THO lesions compared to normal bone tissues. Although our findings provide valuable insights into the molecular mechanisms of THO, we acknowledge some limitations. First, the sample size of this study was relatively small, with three randomly selected from the seven samples collected as a representative patient population for miRNA sequencing, which may limit the representability of our findings. Differences between individuals may affect the generalizability of our results. To address this issue, we used Western blot to verify the expression of key proteins associated with differentially expressed mirnas. Future studies with larger sample sizes and stratified sampling will be essential to further validate our results and explain differences in patient populations. In addition, case-control studies have had limited sample sizes due to the challenges of collecting THO cases, and the distinction between THO tissue and various types of soft tissue has not been studied. The results of this study demonstrate the critical importance of distinguishing the molecular features of THO from normal soft tissue and help elucidate the specific role of DE-miRNAs in THO pathogenesis. Therefore, animal models of THO and cell cultures from THO tissues and normal bone tissues are recommended for future studies to validate our findings and explore the mechanisms by which these mirnas regulate osteogenic differentiation. These experimental approaches could be useful based on our findings of mirna targeting THO key pathways such as BMP, HIF 1 and PI3K/AKT. With further studies, we can deepen our understanding of the underlying mechanisms of THO and provide directions for the development of new therapeutic interventions.
